# Distinct effects of general anesthetics on lung metastasis mediated by IL-6/JAK/STAT3 pathway in mouse models

**DOI:** 10.1038/s41467-019-14065-6

**Published:** 2020-01-31

**Authors:** Ru Li, Yujie Huang, Jun Lin

**Affiliations:** 1 Department of Anesthesiology, Stony Brook University Health Science Center, Stony Brook, NY 11794-8480 USA; 2 School of Dental Medicine, Stony Brook University, Stony Brook, NY 11794 USA

**Keywords:** Metastasis, Metastasis

## Abstract

Metastasis can occur following surgical resection of solid tumors and metastasis is the main cause of cancer death. The role of anesthetics used during surgery in cancer metastasis and the underlying mechanism remains largely unknown. Here we show that surgical dissection of primary tumors in mice under anesthesia with sevoflurane leads to significantly more lung metastasis than with propofol in both syngeneic murine 4T1 and xenograft human MDA-MB-231 breast cancer models. Sevoflurane increases the level of serum IL-6, which activates STAT3 and the infiltration of CD11b+ myeloid cells into the lung. Interruption of IL-6/JAK/STAT3 pathway by a JAK inhibitor AZD1480 reverses the pro-metastatic effect of sevoflurane and the associated increase of both activated STAT3 and infiltrated CD11b+ cells in 4T1 model. Our study provides the preclinical evidence informing the distinct effects of anesthetics on metastasis of breast cancers through change of cytokines and the tumor microenvironment.

## Abstract (web summary)

The effects of anesthetics on cancer metastasis are largely unknown. Here, the authors show in two preclinical mouse models that surgical resection of primary tumours under anesthesia with sevoflurane leads to more lung metastases than with propofol and that such distinct effects are associated with change of cytokines and modulation of myeloid cells in the lung.

## Introduction

Complete resection of solid tumors offers the hope of cure and is often the first-line treatment. However, recurrent metastasis in vital organs does occur and is the major cause of mortality. Despite curative intent of surgery, it has been suggested that the perioperative period presents many risks to cancer patients^1^. An on-going controversy is whether anesthetics used during surgery substantially influence the outcome of cancer patients^2^. Several retrospective studies have suggested that volatile anesthetics, mostly sevoflurane, were associated with worse outcome of patients than intravenous anesthetic propofol after cancer surgery but not confirmed by others^3–7^. The nature of retrospective studies and inconsistent reports precludes the conclusion on the role of anesthetics in patient outcome. Furthermore, the effects of anesthetics in cancer metastasis and potential mechanism remains largely unknown and difficult to be addressed in clinical studies, partially due to the heterogeneity in both patients and diseases. Thus, vigorous preclinical studies are needed to define the role of anesthesia in cancer and to unravel the molecular mechanism of anesthetics in cancer metastasis.

In this study, we compare the effects of sevoflurane to propofol on lung metastases in the syngeneic murine 4T1 and xenograft human breast MDA-MB-231 mouse models, both of which incorporate clinically relevant surgery. The syngeneic murine 4T1 mouse model, which in part resembles the formation of human breast cancers, allows us to perform surgery to mimic mastectomy in patients. The murine mammary carcinoma 4T1 cells are highly tumorigenic and reliably metastasize to multiple distant organs^8,9^. Moreover, the progressive spread of 4T1 metastases to distant organ is very similar to that of human mammary cancer. The micro-metastasis emerges at the early stage of the disease, and then activate to become metastasis over time^10,11^. In this way, there is a window of time to target the early stage of metastasis. MDA-MB-231 cell is an aggressive triple negative breast cancer cell line and is able to colonize lung, bond, and brain^12–14^. Nonobese diabetic (NOD) severe combined immunodeficiency (SCID) mouse is chosen for the successful application of this model to evaluate drug anti-metastases effects^15^. Therefore, these two models are considered to be clinically relevant models for breast cancer metastasis^16^.

In this study, we show that the mice receiving volatile anesthetic sevoflurane during surgical removal of primary tumor develop more lung metastases than those receiving propofol. To delineate the underlying mechanisms, we evaluate the effects of anesthetics on cancer cells, cytokine levels and lung microenvironment, primarily in 4T1 model for the complete immunity of the subject mice. We identify interleukin 6 (IL-6) as the key contributor induced by sevoflurane to prime the lung microenvironment through activation of IL-6/signal transducer and activator of transcription 3 (STAT3) pathway and infiltration of CD11b+ cells. As Janus kinase (JAK) mediates IL-6 on activation of STAT3, we use a JAK2 inhibitor, AZD1480, to target IL-6/JAK/STAT3 pathway and find it notably reverses the lung metastasis and increased CD11b+ cells in lung induced by sevoflurane in the 4T1 model. Our study not only unravel the mechanism underlying sevoflurane-promoted cancer metastasis but also offers a promising intervention to overcome the risk of general anesthesia.

## Results

### Sevoflurane promotes lung metastases in mouse models

**Fig. 1:**
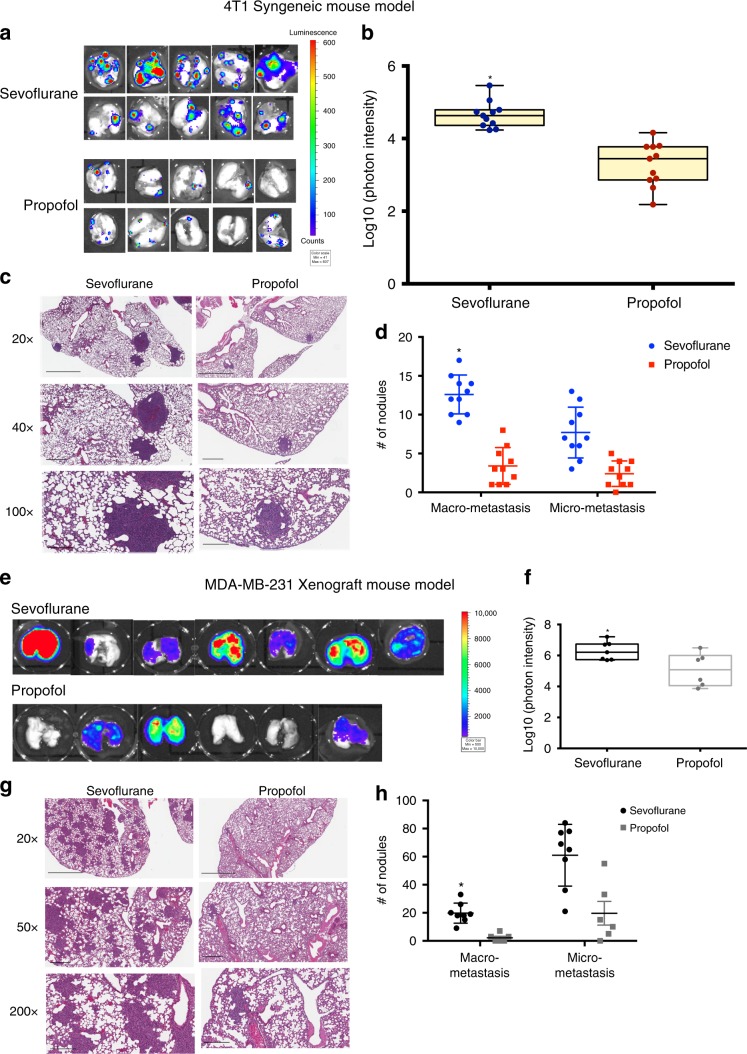
Mice underwent mastectomy with sevoflurane developed more lung metastasis than with propofol. Mice bearing primary tumors were generated by orthotopically implanted with the luciferase-tagged murine 4T1 breast cancer cells or human MDA-MB-231 breast cancer cells in the mammary fat pads in *balb/c* mice or NOD-SCID mice respectively. Surgical dissection of primary tumor with sevoflurane significantly increased lung metastasis than with propofol in both models. Mastectomy was performed in mice models and lung metastasis were evaluated two weeks after surgery. In the 4T1 model, **a** ex vivo lung bioluminescent imaging and **b** photon intensity of them showed remarkably more lung metastasis in the mice received sevoflurane than those received propofol (*n* = 10 for each group, *p* = 0.013, unpaired t-test). Center line of box plot represents the median, bounds represent the first and third quantiles, and whiskers represent the lowest and highest value. The result of bioluminescent imaging was confirmed by (**c**) histology (H&E staining) pictures of the lung sections from each group at different magnifications. The macro-metastatic nodules versus micro-metastatic nodules in each group were quantified. **d** The examination of number and size of metastatic nodules showed that significantly more macro-metastases were observed in sevoflurane group than in propofol group (*n* = 10, *p* = 0.019 for macro-metastasis, two-way ANOVA + Dunnett’s post hoc tests). The results in 4T1 syngeneic model were observed in the MDA-MB-231 xenograft model as shown in (**e**) bioluminescent images of lung area of mice, **f** quantitative measurements of the ex vivo lung photon intensity (*p* = 0.04, *n* = 8 for sevoflurane group and *n* = 6 for propofol group, unpaired *t*-test), **g** histology of the lungs and **h** quantification of metastatic nodules (*n* = 8 for sevoflurane group and *n* = 6 for propofol group, *p* = 0.016 for macro-metastasis, two-way ANOVA + Dunnett’s post hoc tests). Data are shown as mean ± S.D. Scale bar: 20× = 2 mm, 40× = 600 μm, 100× = 300 μm.  Source data are provided as a Source Data file.

Murine 4T1 cells stably expressing luciferase were implanted in unilateral mammary fat pad of *balb/c* mice under inhaled isoflurane. The implantation procedure was done within 10 min to minimize the exposure of mice to isoflurane. When the volume of primary tumor reached around 500 mm^3^, surgical dissection was conducted under inhaled sevoflurane or intraperitoneal (i.p.) injection of propofol and anesthesia were maintained for three hours. Two weeks after surgical removal of primary tumor, the mice received sevoflurane developed remarkably more lung metastases than those received propofol as shown by ex vivo bioluminescent imaging (Fig. 1a, b, *n* = 10, *p* = 0.013, unpaired *t*-test). Histology analysis confirmed the difference in lung metastases (Fig. 1c). Nodule count correlated very well with ex vivo IVIS image (Fig. 1d, *p* = 0.019, two-way ANOVA + Dunnett’s post hoc tests).

To validate the differential effects of anesthetics on lung metastasis observed in the 4T1 model, we employed another mouse model with human MDA-MB-231 breast cancer cells stably expressing luciferase in NOD-SCID mice under identical experimental condition. The difference in lung metastasis between the sevoflurane and propofol groups was significant following mastectomy as measured by ex vivo bioluminescent imaging (Fig. 1e, f, n = 7 for sevoflurane group and *n* = 6 for propofol group, *p* = 0.04, unpaired *t*-test). Consistently, H&E staining showed more metastatic foci in the lungs of mice anesthetized with sevoflurane compared to mice anesthetized with propofol (Fig. 1g, h, *p* = 0.016, two-way ANOVA + Dunnett’s post hoc tests).

Together, we demonstrated that sevoflurane was associated with significant increased lung metastasis than propofol in two models, one with murine breast cancer cells and one with human breast cancer cells. As the next step to delineate the underlying mechanism of the distinct effects of these two most commonly used anesthetics on metastasis, we used 4T1 syngeneic mouse model in the following experiments owing to the complete immunity of the subject mice.

### Multiple exposures of anesthetics have no additive effects

To investigate whether anesthetics have direct effects on tumor cell growth in vivo, sevoflurane or propofol was used during implantation of 4T1 cells into one mammary fat pad of the *balb/c* mice and repeated exposure to same anesthetics for one hour was conducted every 2 days. The growth of primary tumor over two weeks was tracked by measuring the sizes (Supplementary Fig. 1A), weights (Supplementary Fig. 1B), and in vivo bioluminescent imaging (Supplementary Fig. 1C). The growth curve (Supplementary Fig. 1A) and the final primary tumor weight (Supplementary Fig. 1B) have showed no significant difference between sevoflurane and propofol group, which imply that anesthetics did not alter the course of primary tumor growth or both anesthetics have similar effect on the proliferation of 4T1 cells in vivo. Two weeks after implantation, the primary tumor was resected under three-hour anesthesia with the same anesthetics as for implantation and repeated exposures. After that, exposure to the same anesthetics for one hour was continued every two days for two weeks. Two weeks after surgical removal of primary tumor, the mice received sevoflurane developed significantly more lung metastases than those received propofol as showed by in vivo and ex vivo bioluminescent imaging (Supplementary Fig. 1D, E) as well as histology (Supplementary Fig. 1F, G). However, multiple exposures of sevoflurane do not show an additive pro-metastatic effect, compared with single exposure during the surgery (Supplementary Fig. 1H–J). It suggests that some intrinsic factors in surgical phase are needed for sevoflurane to alter the course of metastases.

### Effect of anesthetics on functions of 4T1 cells in vitro

Anesthetics have been suggested to target tumor cells via various cellular pathways, which might affect the cascade of metastasis^17,18^. To explore the direct effects of anesthetics on cancer cell function, we tested sevoflurane and propofol on the viability and migration of 4T1 cells. In these in vitro studies, we chose the relevant clinical dose of sevoflurane (0.2 mM, which is 1.3 MAC), and approximately equivalent clinical dose of propofol (4 µg per ml). Cell viability was measured by MTT assay after 24-h incubation. Sevoflurane did not affect cell viability at concentrations of 0.2 mM or lower but exhibited significant anti-proliferation effect on 4T1 cells at 1 mM or higher (Supplementary Fig. 2A). Propofol failed to inhibit cell proliferation within indicated range of doses (Supplementary Fig. 2B). The migration of 4T1 cells was assessed by wound healing assay at 24 and 48 h. Both sevoflurane and propofol suppressed the migration of 4T1 cells in a dose dependent manner (Supplementary Fig. 2C, D). Thus, the in vitro effects of both anesthetics on 4T1 cells do not seem to echo their distinct in vivo effects, suggesting that anesthesia might change the tumor microenvironment that regulates the seeding and growth of metastatic tumors.

### Sevoflurane activates IL-6/STAT3 pathway in the lung

**Fig. 2:**
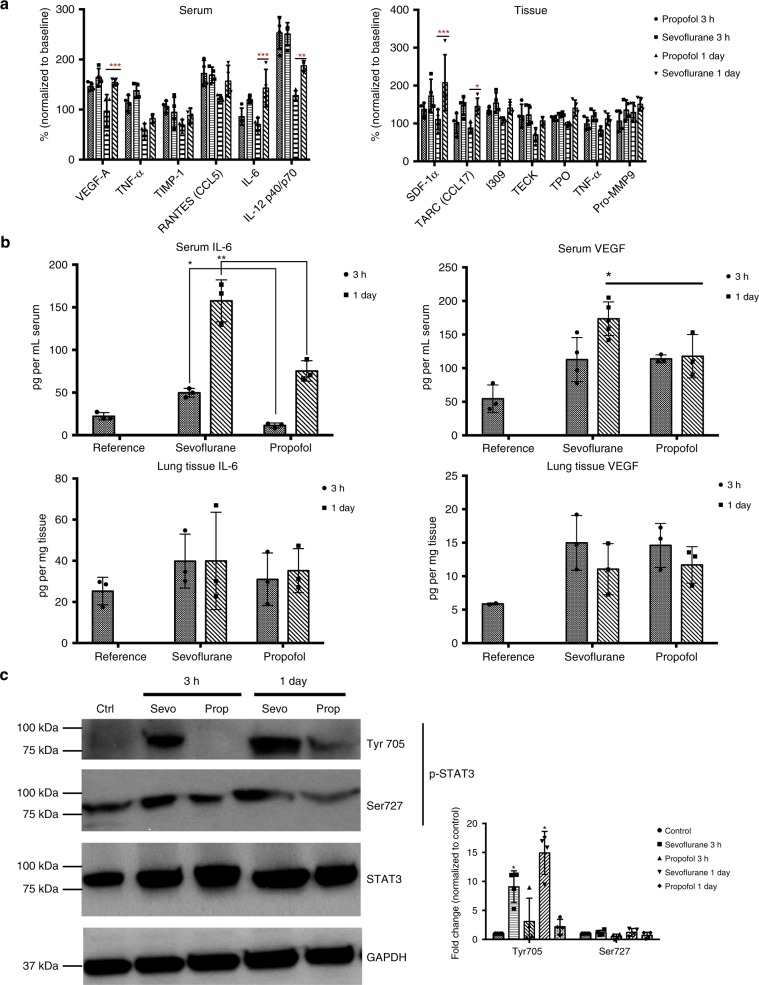
Sevoflurane elevated the mice serum level of IL-6 and activated its downstream effector STAT3 in lung tissues. **a** Anesthetics-induced cytokine changes in lung tissues were evaluated by Cytokine array analysis in the 4T1 syngeneic mouse model. Three hours or one day after mastectomy under sevoflurane or propofol, mice were euthanized to collect serum and lung tissue, which were then subject to cytokine array c-100. Data were normalized against the reference (mouse bearing tumors without surgery) across membranes to calculate relative expression of cytokines in the serum or lung (Serum one day: sevoflurane vs propofol, VEGF-A, *p* = 0.001; IL-6, *p* < 0.001; IL-12, *p* = 0.007. Tissue one day: sevoflurane vs propofol, SDF-1 α, *p* < 0.001; TARC, *p* = 0.023. Two-way ANOVA + Dunnett’s post hoc tests). **b** Serum level of IL-6 was significantly higher in the sevoflurane group compared to the propofol group for both three-hour (*p* = 0.02, two-way ANOVA + Dunnett’s post hoc tests) and one-day after surgery (*p* = 0.002, two-way ANOVA + Dunnett’s post hoc tests) by ELISA analysis of IL-6 and VEGF in serum and lung lysate. Serum VEGF level was significantly higher in the lungs one day after surgery with sevoflurane than with propofol (*n* = 3, *p* = 0.03, two-way ANOVA + Dunnett’s post hoc tests). **c** Sevoflurane treated mice showed significant higher level of p-STAT3 (Tyr 705) in the lungs than propofol at both three-hour (*p* = 0.02, two-way ANOVA + Dunnett’s post hoc tests) and one-day (*p* = 0.01, two-way ANOVA + Dunnett’s post hoc tests) after surgery by Western blot. No significant change in Ser727 was observed (*n* = 3, two-way ANOVA + Dunnett’s post hoc tests). Phosphorylation at Tyr 705 is generally agreed as the main activated form of STAT3. Data presented as the mean ± S.D. Source data are provided as a Source Data file.

We next evaluated the potential change of cytokines associated with these two anesthetics in the early stage of post-surgical metastases. The change of cytokines could unravel the coordinated molecular network in the metastatic process. Since the elimination of anesthetics after several hours’ exposure is usually within one day, we focused the short-term effects of anesthetics on the levels of cytokines/chemokines in serum and lung using 4T1 syngeneic mouse model. Following the same surgical procedures and anesthesia regiment as described above, mouse serum and lung tissue were harvested three hours or one day after mastectomy for secreted cytokine/chemokine profiling. A group of mice inoculated with primary tumors with no surgery and no anesthesia served as the reference. As shown in Fig. 2a and Supplementary Fig. 3, we found that sevoflurane increased pro-inflammatory cytokines known to facilitate mammary tumor metastasis, such as IL-6, interleukin-12, vascular endothelial growth factor (VEGF), stromal cell-derived factor 1 (SDF-1 α), TARC (CCL17), chemokine ligand 1 (I-309/CCL1), and pro-matrix metallopeptidase 9 (Pro-MMP9) in comparison to reference ^19^. In opposite to sevoflurane, propofol decreased pro-inflammatory cytokines/chemokines including tumor necrosis factor (TNF-α).

We chose to validate the change of IL-6 and VEGF for their key roles in cancer metastasis and association with metastatic breast cancers^20^. Serum and lung lysate obtained from the mice received sevoflurane or propofol were subject to ELISA assay of IL-6 and VEGF. Consistent with results obtained from cytokine array, the serum level of IL-6 significantly increased in sevoflurane group, but not in propofol group, three hours after surgery (Fig. 2b, *p* = 0.02, two-way ANOVA + Dunnett’s post hoc tests). One day after surgery, the IL-6 level in sevoflurane group was two times of propofol group (*p *= 0.002, two-way ANOVA + Dunnett’s post hoc tests). Meanwhile, the IL-6 level in lung did not significantly change after surgery in both groups (Fig. 2b). Similarly, serum level of VEGF also increased in mice treated with sevoflurane compared to propofol with statistical significance obtained at one day after surgery (*p* = 0.03, two-way ANOVA + Dunnett’s post hoc tests). No significant change of VEGF was observed in mice lung (Fig. 2b). Based on ELISA results, sevoflurane induced more remarkable change of IL-6 than VEGF. This led to our further analysis of the IL-6 signaling pathway.

IL-6 predominantly regulates STAT3-mediated oncogenic processes through JAK^21^. Elevated IL-6 activates its downstream effectors thus we expected a higher level of p-STAT3 in sevoflurane group than propofol group. The expression pattern of the phosphorylated forms of STAT3, p-Tyr705 and p-Ser727, was examined in the lung tissues by Western blot. Indeed, sevoflurane group had significantly higher levels of p-STAT3 (p-Tyr705) than propofol group at three hours after surgery (Fig. 2c, *p* = 0.02, two-way ANOVA + Dunnett’s post hoc tests), and the difference increased at one day after surgery (*p* = 0.01, two-way ANOVA + Dunnett’s post hoc tests). No significant change was observed in p-Ser727 for both groups and time points. IL-6 mainly activates STAT3 through phosphorylation of Tyr705^22^. The regulation of STAT3 by phosphorylation of Ser727 is controversial as both enhancing and repressing STAT3 activity were reported^23–25^. Thus, increased p-Tyr705 but not p-Ser727 agrees well with our hypothesis that sevoflurane activates IL-6/JAK/STAT3 pathway in mice under surgical conditions.

### AZD1480 alleviates sevoflurane-promoted lung metastasis

**Fig. 3:**
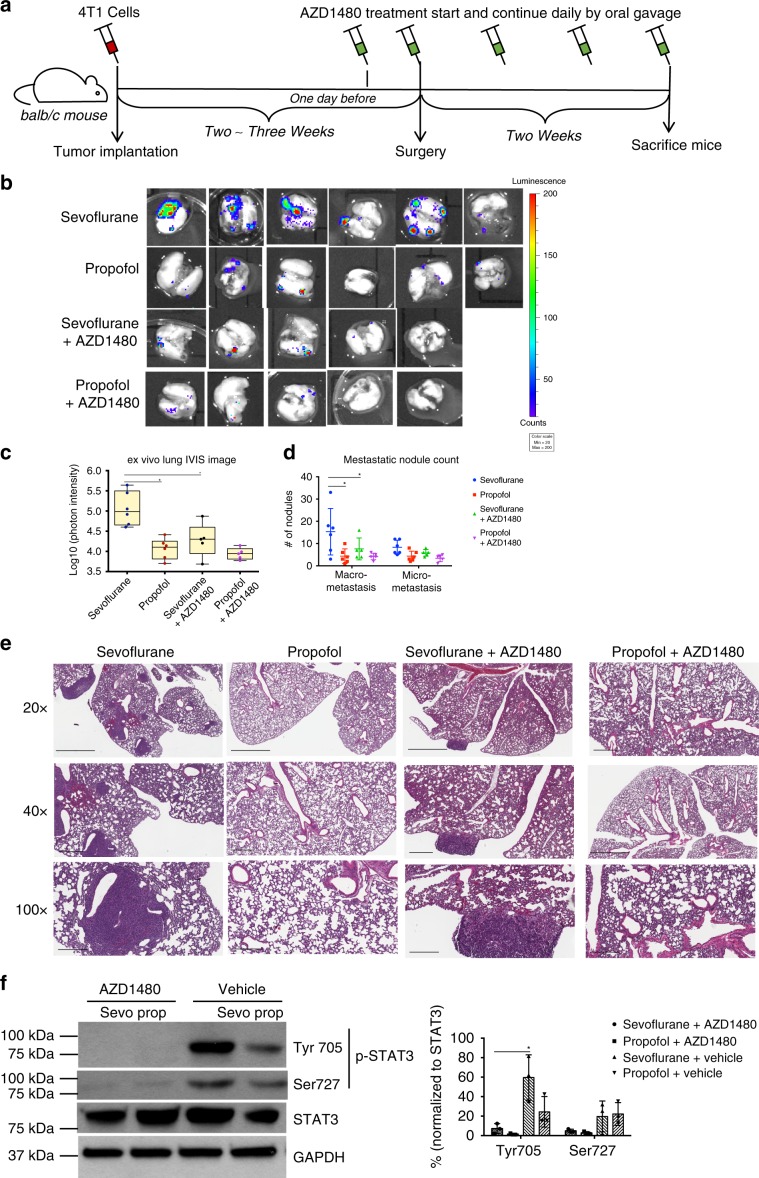
AZD1480 alleviated sevoflurane-promoted lung metastasis through blocking IL-6/JAK/STAT3 pathway. **a** In the 4T1 mouse model, tumor bearing mice were orally administrated with vehicle or AZD1480 one day before surgery and the administration continued once a day until the end of the experiment. **b** Bioluminescent image of ex vivo lung and **c** quantitative photon intensity of lung were used to measure the metastatic burdens. Again, sevoflurane significantly promoted lung metastasis than propofol (*p* = 0.002, one-way ANOVA + Tukey post hoc test). Treatment with AZD1480 remarkably reduced sevoflurane-promoted lung metastasis (*n* = 6 for groups treated with vehicle and *n* = 5 for groups treated with AZD1480, *p* = 0.003, one-way ANOVA + Tukey post hoc test). The line within each box represents the median. Upper and lower edges of each box represent the first and third quantiles. The whiskers represent the lowest and highest value. **d** Quantification of metastatic nodule based on (**e**) lung histology confirmed the bioluminescent analysis with significant more macro-metastatic nodules in sevoflurane group compared to propofol group or AZD1480 group (*n* = 6 for groups treated with vehicle and *n* = 5 for groups treated with AZD1480; sevoflurane vs propofol, *p* = 0.008; sevoflurane vs sevoflurane + AZD1480, *p* = 0.03; two-way ANOVA + Dunnett’s post hoc tests;). Scale bar: 20× = 2 mm, 40× = 600 μm, 100× = 300 μm. **f** AZD1480 treatment significantly reduced the phosphorylated STAT3 (p-Tyr705) level in the lungs of sevoflurane group by Western blot analysis (*n* = 6 for groups treated with vehicle and *n* = 5 for groups treated with AZD1480, *p* = 0.015, two-way ANOVA + Dunnett’s post hoc tests). Data are shown as the mean ± S.D. Source data are provided as a Source Data file.

To confirm the role of IL-6/JAK/STAT3 pathway in mediating sevoflurane-enhanced lung metastases, we then tested the effect of interrupting the IL-6/JAK/STAT3 signaling cascade with a JAK inhibitor AZD1480 in 4T1 model. Prior to the surgery, mice were dosed by oral gavage once with vehicles in the control group or AZD1480 in the treatment group. Anesthesia and surgery followed with the above protocol. After surgery, mice were treated with vehicles or AZD1480 once a day for two weeks (Fig. 3a). Through this experiment, AZD1480 notably reduced the lung metastases in the mice received sevoflurane, evidenced by ex vivo bioluminescent imaging (sevoflurane vs propofol, *p* = 0.002; sevoflurane vs sevoflurane + AZD1480, *p* = 0.003; one-way ANOVA + Tukey post hoc test) and nodule counting (sevoflurane vs propofol, *p* = 0.008; sevoflurane vs sevoflurane + AZD1480, *p* = 0.03; two-way ANOVA + Dunnett’s post hoc tests) (Fig. 3b–e). No significant difference was observed in propofol groups with treatment of AZD1480 or vehicle. To further confirm that AZD1480 interferes with IL-6/JAK/STAT3 pathway, phosphorylation level of downstream STAT3 was analyzed by Western blot. AZD1480 notably reduced phosphorylation level of STAT3 (p-Tyr705) in the lung of mice two weeks after surgery under sevoflurane (Fig. 3f, *p* = 0.015, two-way ANOVA + Dunnett’s post hoc tests). AZD1480 also reduced p-STAT3 in propofol group but without significance.

**Fig. 4:**
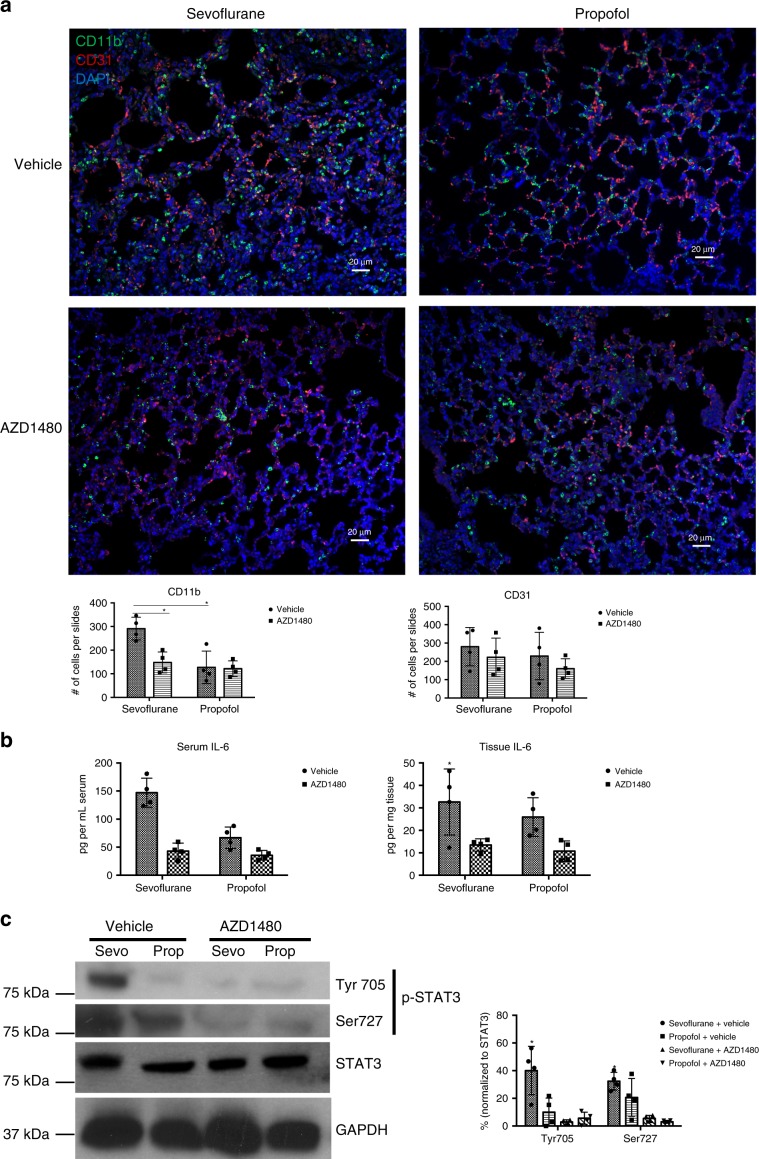
AZD1480 treatment reduced the infiltration of CD11b + myeloid cells associated with sevoflurane in the lung. **a** Sevoflurane increased CD11b+ cells in the lung one day after surgery compared to propofol by immunofluorescence imaging. Treatment with AZD1480 significantly reduced infiltrated CD11b+ cells (green) induced by sevoflurane in the lungs (*n* = 4 mice in each group; sevoflurane vs propofol, *p* = 0.007; sevoflurane vehicle vs AZD1480, *p* = 0.032; two-way ANOVA + Dunnett’s post hoc tests, Scale bar = 20 μm). No significant change was observed with CD31 positive cells (red). One day surgery, lungs of the mice were sectioned and stained with anti-CD11b, anti-CD31, and DAPI. **b** One day after surgery, AZD1480 treatment dramatically reduced serum and lung tissue level of IL-6 (*n* = 4, *p* = 0.001 and 0.02 respectively, two-way ANOVA + Dunnett’s post hoc tests) by ELISA assays. **c** Western blot analysis of STAT3 phosphorylation showed that AZD1480 treatment significantly reduced p-Tyr705 (sevoflurane vs propofol *p* = 0.03, sevoflurane vs AZD1480 *p* = 0.001, two-way ANOVA + Dunnett’s post hoc tests) and p-Ser727 (sevoflurane vs AZD1480 *p* = 0.001, *n* = 4, two-way ANOVA + Dunnett’s post hoc tests) one day after surgery with sevoflurane. Data presented as the mean ± S.D. Source data are provided as a Source Data file.

The tumor microenvironment in lung is composed of a complex network of stroma cells. In mouse models of breast cancers, CD11b+ myeloid cells have been reported as a major contributor to establish pre-metastatic niche in the lung^26,27^. To corroborate the notion that sevoflurane affects CD11b+ cells in the lung, we performed the immunofluorescence analysis of mouse lung in both 4T1 and MDA-MB-231 models. Indeed, we have observed increased CD11b+ cells (green) in lung tissues in the mice under sevoflurane compared to propofol in syngeneic 4T1 model one day after surgery (Fig. 4a, *p* = 0.007, two-way ANOVA + Dunnett’s post hoc tests). Sevoflurane-induced infiltration of CD11b+ cells was also confirmed in xenograft MDA-MB-231 mouse model (Supplementary Fig. 4). Since AZD1480 reversed sevoflurane-promoted metastasis, we then hypothesized that blockage of IL-6/JAK/STAT3 pathway by AZD1480 would also lead to the reversal of the infiltration of CD11b+ cells. Indeed, AZD1480 significantly reduced the infiltration of CD11b+ cells in the lung of mice received sevoflurane one day after surgery (Fig. 4a, *p* = 0.032, two-way ANOVA + Dunnett’s post hoc tests). We also examined CD31+ cells (red) for potential change of vasculature but found no difference between sevoflurane and propofol with or without AZD1480.

The sevoflurane-induced enrichment of CD11b+ cells in the pre-metastatic lung is dependent on IL-6/JAK-STAT3 signaling axis, since AZD1480 also significantly inhibited the sevoflurane-induced increase of IL-6 (Fig. 4b; serum, sevoflurane vehicle vs AZD1480, *p* = 0.001; lung tissue, sevoflurane vehicle vs AZD1480, *p* = 0.02; two-way ANOVA + Dunnett’s post hoc tests) and the activation of STAT3 one day after surgery (Fig. 4c, p-Tyr705, sevoflurane vs propofol *p* = 0.03, sevoflurane vs AZD1480 *p* = 0.001; p-Ser727, sevoflurane vs AZD1480 *p* = 0.00; two-way ANOVA + Dunnett’s post hoc tests).

## Discussion

Although several large prospective clinical trials are ongoing in this area, convincing clinical evidence regarding the choice of general anesthetics related to cancer patient outcome is still lacking. The large clinical trials involving common cancer types are not due to be completed for years and may be still inconclusive due to the heterogeneity in both the patient and the disease. Our study demonstrates the distinct effects of two modern general anesthetics in breast cancer metastasis in two preclinical animal models. We acknowledge that animal studies may not always be translatable directly to humans. Nevertheless, it represents a step toward the evidence-based choice of anesthetics in breast cancer patients.

The mechanism of anesthetics in the metastatic process has been poorly understood and challenging to study due to lack of appropriate preclinical models and broad spectrum of anesthetic actions. We incorporated surgery into two mouse models to decipher the role of anesthetics in this process. Collectively, our data suggests that volatile anesthetic sevoflurane promotes lung metastases through eliciting the expression of IL-6 and enriching CD11b+ myeloid cells in pre-metastatic lung during the perioperative period. The alleviation of sevoflurane-promoted lung metastases by the JAK inhibitor AZD1480 indicates a potential therapeutic approach that merits further investigation.

Anesthetics could affect the course and growth of metastatic tumors by changing the cancer cells, the tumor microenvironment, or both. Both anesthetics inhibited cancer cell migration without interference of cell viability in vitro. Furthermore, multiple exposures of the anesthetics did not affect the primary tumor growth and did not have additive effect on the lung metastasis. Therefore, it suggests that anesthesia most likely changes the tumor microenvironment that regulates metastasis in the context of surgery. Through screening with protein array and validating by ELISA, we found that sevoflurane-enhanced lung metastasis through increasing the expression of pro-inflammatory cytokines. Pro-inflammatory cytokines are the essential regulators in cancer metastatic process^28,29^. Among various targets identified in cytokine array, IL-6 is a potent inflammatory cytokine that supports angiogenesis, modulation of inflammation, and cancer cell evasion from immune surveillance in the tumor microenvironment^30,31^. High IL-6 serum levels are associated with poor prognosis in breast cancer patients^20^. Moreover, general anesthetics have been shown to affect several key inflammatory cytokines including IL-6^32^. JAK-mediated STAT3 tyrosine phosphorylation compromises the antitumor immunity in tumor microenvironment and promotes the malignant cell proliferation and survival^33–35^. Our data reveal that the elevated IL-6 level with sevoflurane leads to the activation of IL-6/JAK/STAT3 pathway as evidenced by the increase of phosphorylation on STAT3. Subsequently, the inhibition of IL-6/JAK/STAT3 by AZD1480 successfully attenuated lung metastasis promoted by sevoflurane.

The growth and metastasis of cancer cells largely hinge on a favorable microenvironment at the distant site. Tumor-associated macrophages (TAM), myeloid-derived suppressor cells (MDSC), and microvascular proliferations contribute significantly to the formation of pre-metastatic niche and subsequent growth of metastatic tumors^36,37^. CD11b is expressed in many leukocytes including monocytes and macrophages. CD11b+ cells are the main components in tumor microenvironment that increased in the metastatic lung. We show that the accumulation of CD11b+ myeloid cells before cancer cell infiltration was manifested in the lung shortly after surgery with sevoflurane anesthesia. We further show that reduction of STAT3 phosphorylation by AZD1480 reversed the enriching CD11b+ myeloid cells in pre-metastatic lung during postoperative period. Thus, the accumulation of CD11b+ cells into lung induced by sevoflurane as shown in both mouse models suggests a distinct effect of sevoflurane over propofol in priming the lung microenvironment for metastatic tumors.

In summary, in this report we show that a volatile anesthetic sevoflurane leads to more lung metastasis than an intravenous anesthetic propofol following surgery in two mouse models of spontaneous metastasis. We further show the distinct effects of anesthetics on the production of pro-inflammatory cytokines including IL-6 and homing the pre-metastatic lung microenvironment. The alleviation of sevoflurane-promoted lung metastasis by IL-6/JAK/STAT3 inhibitor AZD1480 indicates a potential therapeutic target that merits further investigation. Our results can be used toward evidence-based medicine for anesthesia care of cancer patients.

## Methods

### Ethics statement

All of the animals used in the experimental procedures were approved by the Institutional Animal Care and Use Committee (IACUC) at Stony Brook University (917821), purchased from the Jackson Laboratory (Boston, MA), and maintained in accordance with federal guidelines. All animals were housed in sterilized plastic cages under pathogen-free conditions (21–25 °C, 12/12 light/dark cycle). Food and water were offered ad libitum. All efforts were made to ameliorate the suffering of the animal during euthanasia using CO_2_ overdose followed by cervical dislocation.

### Animal models and surgical procedures

Female *balb/c* mice were subjected to orthotopically implantation of 4T1 cells (2 × 10^5^ cells per mice) in mammary fat pad. The 4T1 cells were engineered for stable expression of firefly luciferase (provided by Dr, Cia-Hsin Chan, Department of Pharmacological Science, commercial lentiviral vector pTRIPZ encoding firefly luciferase). Implantations were conducted with 2% isoflurane under 10 min. The development of 4T1 tumors was monitored noninvasively by bioluminescent IVIS (IVIS Lumina III, PerkinElmer, Waltham, MA), and the volume of tumors was measured by a calliper on weekly basis and calculated using the formula *V* = (Width^2^ × Length) × 2^−^^1^. When the volume of primary tumor reached around 500 mm^3^, mice with size-matched tumors were randomly assigned to three groups. Two groups receive a surgery to dissect the primary tumor under three-hour anesthesia with sevoflurane or propofol; while the third group did not receive surgery nor anesthesia as reference. During surgery, the delivery of sevoflurane was maintained by a SomnoSuite System, and all groups were monitored with a PhysioSuite with pulse oximeter for oxygen saturation and heart rate and placed on the warming pad for temperature control with SomnoSuite. Propofol was administered by an i.p. injection of 200 mg per kg initially to achieve the surgical condition without significant respiratory depression. Additional injection of propofol (50 mg per kg) was administered to maintain surgical anesthesia level for three hours. Two weeks later after surgery, lung metastasis was monitored by using noninvasive bioluminescent images, and the mice were euthanized to harvest lungs for ex vivo bioluminescent imaging, macroscopic and histological analysis.

For multiple exposure experiment, 4T1 cells were orthotopically implanted in the mammary fat pad of *balb/c* mouse under anesthesia receiving propofol or sevoflurane for roughly 30 min and mice were grouped according to the anesthetics used. After that, mice in each group were treated with corresponding anesthetics for one hour on every two days. When primary tumor reached 500 mm^3^, surgery was performed as described above. After surgery, exposure to anesthetics was continued every two days until the end of experiments. Lung metastatic burden was measured by the method described above.

In NOD-SCID model, primary tumors were generated by orthotopically implantation of MDA-MB-231 cells (5 × 10^5^ cells), which were engineered for stable expression of firefly luciferase (provided by Dr. Cia-Hsin Chan, Department of Pharmacological Science, commercial lentiviral vector pTRIPZ encoding firefly luciferase), into the mammary gland of NOD-SCID mice. Surgical implantation was conducted with 2% isoflurane under 10 min. When the volume of primary tumor reaches around 500 mm^3^, mice were randomly assigned to undergo resection of primary tumor under sevoflurane or propofol for three hours as described above. After surgery, lung metastasis was tracked and quantified by noninvasive bioluminescent imaging. Two weeks later, the mice were euthanized, and the lungs were harvested for ex vivo bioluminescent imaging, macroscopic and histological analysis.

For the experiments with AZD1480, implantation of 4T1 tumor was the identical as described above. The mice were randomly divided into a control group and a treatment group, which were dosed orally with vehicle or AZD1480 (50 mg per kg) respectively. The mice were randomly assigned to receive sevoflurane or propofol for three hours during resection of primary tumors as described above. After surgery, mice were treated by oral gavage once a day for two weeks. At the end of the treatment, mice were euthanized to collect lungs for ex vivo bioluminescent imaging, macroscopic and histological analyses.

### Hematoxylin and eosin (H&E) staining and nodule counting

Mouse lungs were rinsed in phosphate buffered saline to remove blood and then fixed in 4% paraformaldehyde overnight at 4 °C. Tissues were embedded in paraffin and a sampling of sections were taken across lung in the following manner. Two consecutive 5 μm sections were taken and then a number of consecutive 5 μm sections were discarded (typically 20–40 depending on the size of the tumor nodule) before collecting another two consecutive 5 μm sections. This process was repeated along the entire lung. The consecutive sections were then stained using H&E. Metastatic nodules were counted on each H&E paraffin section under a phase contrast microscope and the sum of microscopic counting were taken as the final number of lung metastatic nodules.

### Cytokine array analysis

Following the identical surgical procedures and anesthesia regimens as discussed above, the short-term effects of anesthetics on molecular network in tumor microenvironment were explored. Three hours and one day after mastectomy, mouse serum and lung tissue lysates were collected and probed for secreted cytokine profiling by RayBiotech mouse cytokine array c-1000 according to the manufacturer’s instructions.

### ELISA assay

Mouse blood was harvested for serum and lung were digested with lysis buffer (T-PER Tissue Protein Extraction Reagent, Thermo Fisher Scientific, Waltham, MA, 78510) containing protease inhibitors (cOmplete^TM^ Protease Inhibitor Cocktail and PhosSTOP phosphatase inhibitor, Roche, Pleasanton, CA, 04693159001 and 4906845001). The protein concentration of tissue lysate was determined by BCA Assay (Pierce BCA Protein Assay, Thermo Fisher Scientific, Waltham, MA, 23225). Serum and tissue lysate were subjected to IL-6 and VEGF ELISA assay according to manufacturer’s instruction (Mouse IL-6 DuoSet, Mouse VEGF DuoSet, R&D Systems, Minneapoli, MN, DY406-05, DY493-05). The IL-6 concentrations in serum and lung were calculated according to the volume of serum and the protein concentration of lung tissue lysate respectively.

### Western blot analysis

Lung lysate obtained from mice as described above were subjected to western blot analysis. Lung lysate (30 μg) were resolved by SDS-PAGE on a 10% gel and transferred to a methanol-activated PVDF membrane. For detection of p-STAT3, STAT3, and GAPDH, rabbit monoclonal anti-Phospho-Stat3 (Tyr705, 1:1000, Cell Signaling, 9145), rabbit monoclonal anti-Phospho-Stat3 (Ser727, 1:1000, Cell Signaling, 9134), rabbit monoclonal anti-Stat3 (1:1000, Cell Signaling, 30835), and rabbit monoclonal anti-GAPDH (1:1000, Santa Cruz, sc-32233) were used, followed by the goat anti-rabbit IgG-HRP secondary antibody (1:5000, Santa Cruz, sc-2004). Antibody binding was visualized with enhanced chemiluminescence.

### Immunofluorescence staining

The primary lung tissues were collected, fixed with 4% paraformaldehyde, processed, and paraffinized. Three sections from each lung were stained for infiltrated macrophage (CD11b, Abcam, Cambridge, MA, ab133357, 1:200) and endothelial cells (CD31, BD Pharmingen, San Diego, CA, 55074, 1:100). In brief, the sections were de-paraffinized, retrieved by boiling, blocked with blocking buffer containing normal goat serum, and incubated with primary antibody overnight at 4 °C. After being washed with PBS, the sections were incubated with fluorescent-labeled secondary antibody (Invitrogen, Carlbad, CA), and visualized using ZEISS Axioplan 2 fluorescent microscopy coupled to AxioCam HRm camera. Five randomly selected areas were acquired at ×40 magnification from each section and processed with AxioVision Rel (Version 4.6). All Images shown are representative of four independent experiments. The quantitative analysis of CD11b positive cells was performed by counting CD11b positive products with nuclear staining inside, and the endothelial cells by counting CD31 positive products.

### Statistical analysis

Means and standard deviations are shown in the figures. Unpaired t-test and ANOVA were used to assess significance (*p* < 0.05). Dunnett’s post hoc tests were used to test difference between groups. GraphPad Prism (version 6) was used to calculate statistics.

### Reporting summary

Further information on research design is available in the Nature Research Reporting Summary linked to this article.

## Supplementary information

Supplementary Information

Reporting Summary

## Data Availability

Data generated from this study are included in this article and its Supplementary Information files or will be provided from the corresponding author upon reasonable request. The source data underlying Figs. 1b, d, f, h, 2, 3c, d, f, and 4, and Supplementary Figs. 1A, B, E, G, H-J, 2, 3, and 4 are provided as a Source Data file.

## Source data

Source Data
