# Supplementary material for: Distinct effects of general anesthetics on lung metastasis mediated by IL-6/JAK/STAT3 pathway in mouse models
Source: Nat Commun. 2020 Jan 31;11:642. doi: 10.1038/s41467-019-14065-6 (PMC6994546; doi:10.1038/s41467-019-14065-6)
Supplement: Supplementary file 1 — Supplementary Information [file 41467_2019_14065_MOESM1_ESM.pdf]

Supplementary Information

Distinct effects of general anesthetics on lung metastasis mediated by IL-6/JAK/STAT3 pathway in mouse models

Li, et al

Supplementary Fig. 1

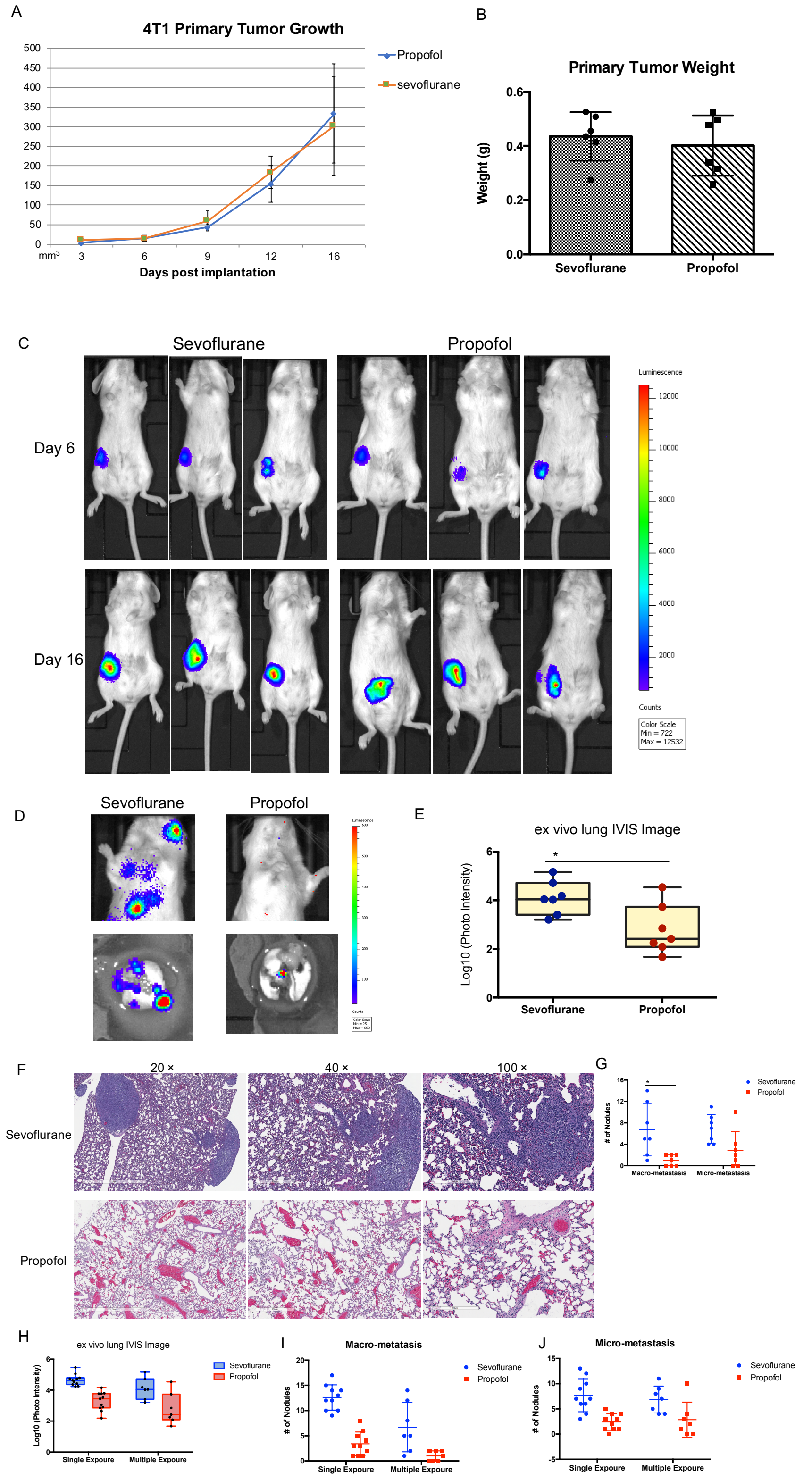

Supplementary Figure 1. Effect of multiple exposure to anesthetics on primary tumor growth and lung metastasis in 4T1 syngeneic mouse model. Repeated exposure to sevoflurane or propofol during and

after implantation does not affect the growth of primary tumor. Surgical dissection of primary tumor under sevoflurane significantly increased lung metastasis than under propofol. **(A)** Tumor volume was measured every two days after exposure to anesthetics (n = 7 for each group, two-way ANOVA test). **(B)** Tumors harvested at day 16 were weighted but no statistical difference between the groups (n = 7 for each group, unpaired t-test). **(C)** Representative bioluminescence imaging on Day 6 and Day 16. **(D)** Representative bioluminescent images obtained from living mice two weeks after surgery with either sevoflurane or propofol plus *ex vivo* bioluminescent imaging of lungs. **(E)** Quantification of photon intensity of bioluminescence of lungs (n = 7 for each group, p = 0.0164, unpaired t-test). Center line of box plot represents the median, bounds represent the first and third quantiles, and whiskers represent the lowest and highest datum within 1.5 × the interquartile range of the lower and upper quantiles. **(F)** Different magnifications of histology of lung indicate the number and size metastatic nodules from spontaneous metastasis. **(G)** The macro-metastatic nodules (diameter > 100 μm) versus micro-metastatic nodules (diameter < 100 μm) in each group were quantified. Significantly more macro-metastasis was observed in sevoflurane group (n = 7 for each group, p = 0.0124 for macro-metastasis, two-way ANOVA + Dunnett's post hoc tests). No significant difference was observed between multiple exposure and single exposure as in ex vivo IVIS signal (H), macro-metastasis (I), and micro-metastasis (J) (n = 10 for single exposure, n = 10 for multiple exposure, two-way ANOVA + Dunnett's post hoc test). Data presented as the mean ± S.D. Source data are provided as a Source Data file.

Supplementary Fig 2

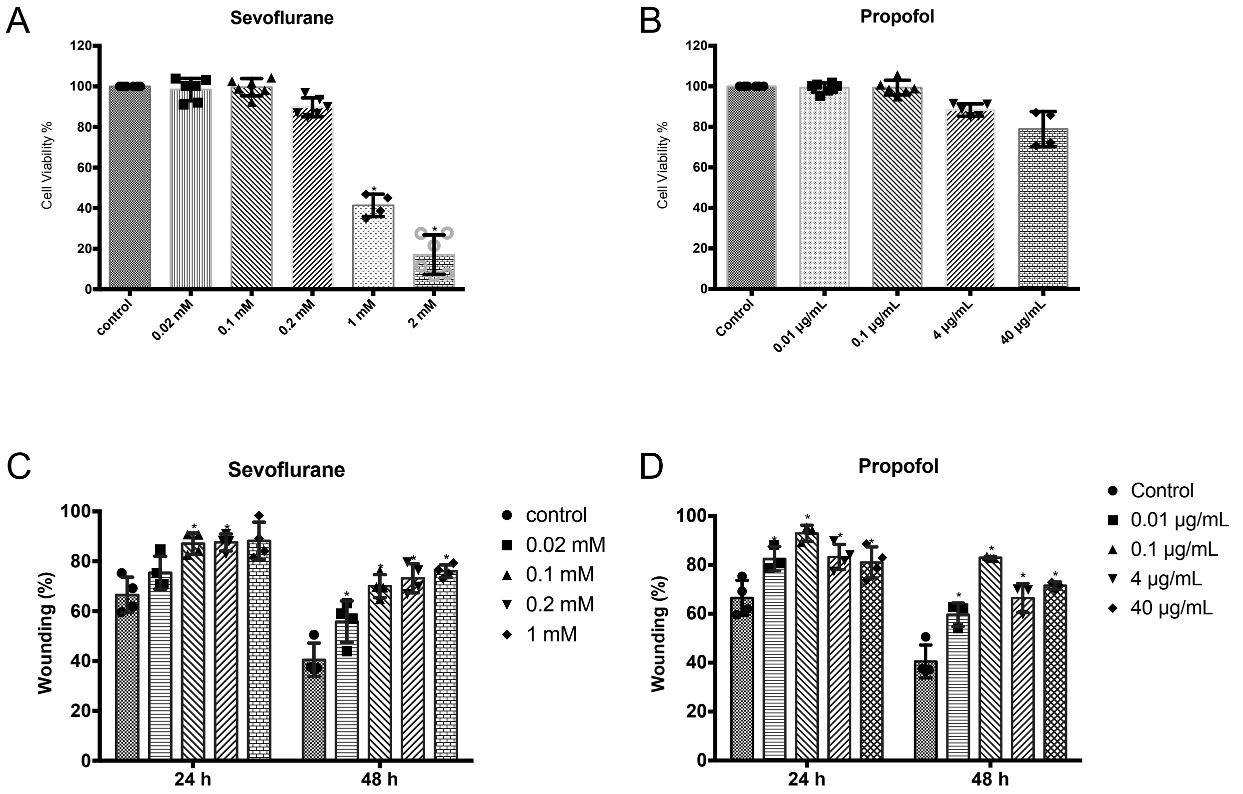

Supplementary Figure 2. Effect of propofol and sevoflurane on the viability and migration of 4T1 cells. The viability of 4T1 cells treated with indicated doses of sevoflurane (A) or propofol (B) were measured by MTT assay. Absorbance at OD490 were quantified and showed. Migration of 4T1 cells were evaluated by wound healing assay. The confluent 4T1 cells was scraped and subjected to indicated doses of sevoflurane (C) or propofol (D). The wound closure was measured in 24 and 48 hours (n = 6, *$p < 0.05$, one-way ANONA + Tukey post hoc test for cell viability assay, two-way ANOVA + Dunnett's post hoc tests for cell migration). Data are shown as the mean ± S.D. Source data are provided as a Source Data file.

Supplementary Fig. 3

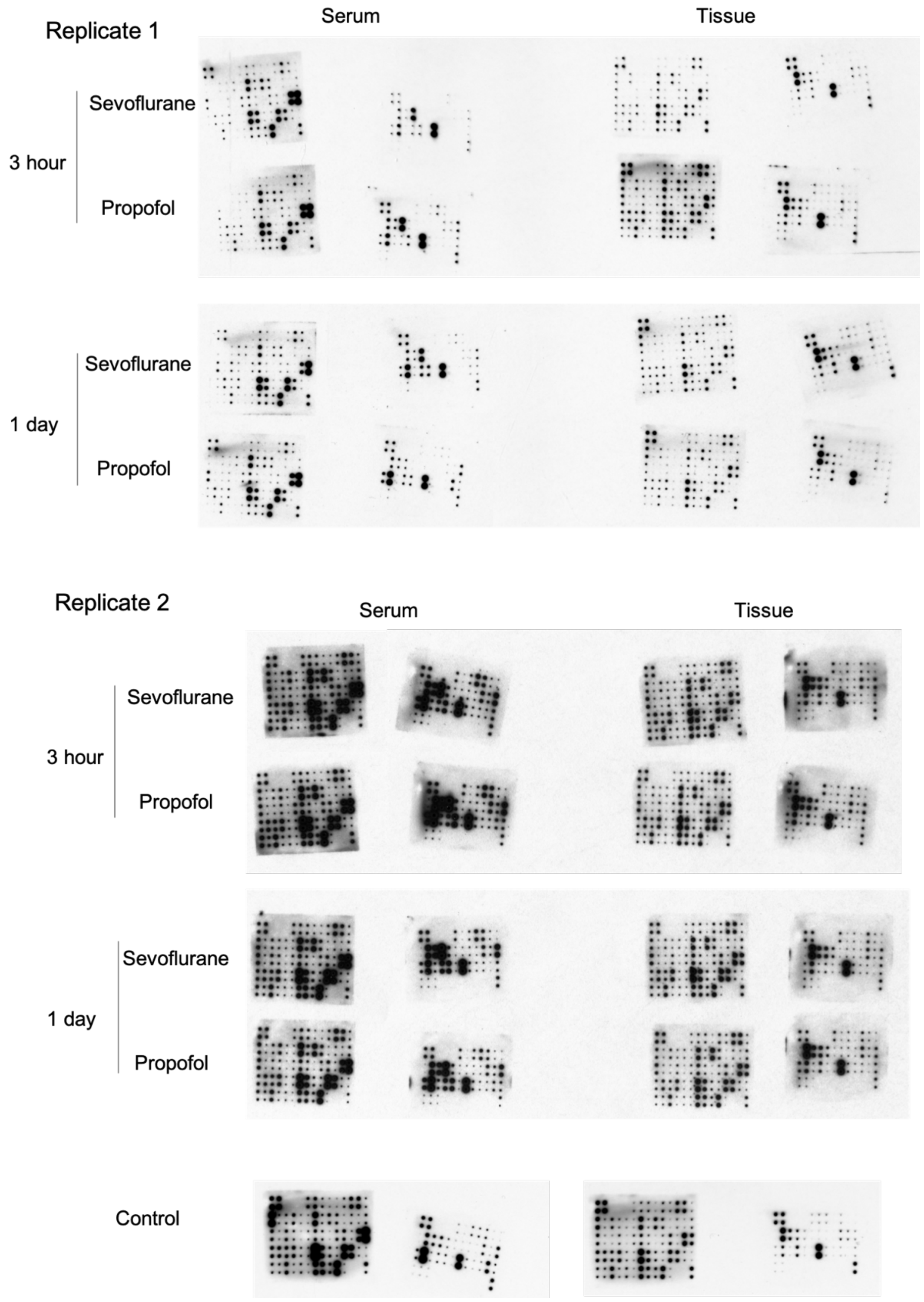

Supplementary Figure 3. Original image of cytokine array c-1000 member showed the intensity of secreted cytokine/chemokine in mouse serum or lung after surgery. Two mice were euthanized for each time point for two biological replicates.

Supplementary Fig. 4

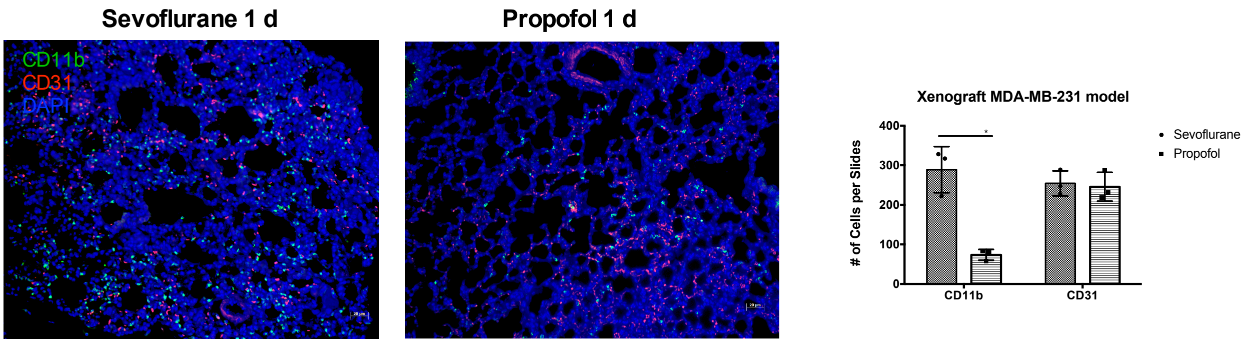

Supplementary Figure 4. Immunofluorescence images of mice lung one day after surgery with sevoflurane or propofol in the xenograft mouse model. Increased CD11b+ cells in mice lung was observed one day after surgery with sevoflurane than propofol. Lungs of the mice were sectioned and stained with anti-CD11b (green), anti-CD31 (red), and DAPI (blue) ($n = 4$ mice in each group, $p = 0.002$, one-way ANOVA + Tukey post hoc test, Scale bar = 20 μm). Data presented as the mean ± S.D. Source data are provided as a Source Data file.
