# Supplementary material for: Distinct effects of general anesthetics on lung metastasis mediated by IL-6/JAK/STAT3 pathway in mouse models
Source: Nat Commun. 2020 Jan 31;11:642. doi: 10.1038/s41467-019-14065-6 (PMC6994546; doi:10.1038/s41467-019-14065-6)
Supplement: Supplementary file 2 — Reporting Summary [file 41467_2019_14065_MOESM2_ESM.pdf]

nature research

Corresponding author(s): Jun Lin

Reporting Summary

Nature Research wishes to improve the reproducibility of the work that we publish. This form provides structure for consistency and transparency in reporting. For further information on Nature Research policies, see Authors & Referees and the Editorial Policy Checklist.

Statistical parameters

When statistical analyses are reported, confirm that the following items are present in the relevant location (e.g. figure legend, table legend, main text, or Methods section).

n/a	Confirmed	
☐	☒	The exact sample size (n) for each experimental group/condition, given as a discrete number and unit of measurement
☐	☒	An indication of whether measurements were taken from distinct samples or whether the same sample was measured repeatedly
☐	☒	The statistical test(s) used AND whether they are one- or two-sided<br>*Only common tests should be described solely by name; describe more complex techniques in the Methods section.*
☐	☒	A description of all covariates tested
☐	☒	A description of any assumptions or corrections, such as tests of normality and adjustment for multiple comparisons
☐	☒	A full description of the statistics including central tendency (e.g. means) or other basic estimates (e.g. regression coefficient) AND variation (e.g. standard deviation) or associated estimates of uncertainty (e.g. confidence intervals)
☐	☒	For null hypothesis testing, the test statistic (e.g. F, t, r) with confidence intervals, effect sizes, degrees of freedom and P value noted<br>*Give P values as exact values whenever suitable.*
☒	☐	For Bayesian analysis, information on the choice of priors and Markov chain Monte Carlo settings
☒	☐	For hierarchical and complex designs, identification of the appropriate level for tests and full reporting of outcomes
☒	☐	Estimates of effect sizes (e.g. Cohen's d, Pearson's r), indicating how they were calculated
☐	☒	Clearly defined error bars<br>*State explicitly what error bars represent (e.g. SD, SE, CI)*

Our web collection on statistics for biologists may be useful.

Software and code

Policy information about availability of computer code

Data collection	IVIS Lumina III (Perkin Elmer), ZEISS Axioplan 2 fluorescent microscopy coupled to AxioCam HRm camera
Data analysis	Axiovision Rel (Version 4.6), Image J (1.47), GraphPad Prism (Version 6)

For manuscripts utilizing custom algorithms or software that are central to the research but not yet described in published literature, software must be made available to editors/reviewers upon request. We strongly encourage code deposition in a community repository (e.g. GitHub). See the Nature Research guidelines for submitting code & software for further information.

Data

Policy information about availability of data

All manuscripts must include a data availability statement. This statement should provide the following information, where applicable:

- Accession codes, unique identifiers, or web links for publicly available datasets
- A list of figures that have associated raw data
- A description of any restrictions on data availability

The datasets generated during and/or analysed during the current study are available from the corresponding author on reasonable request.

Field-specific reporting

Please select the best fit for your research. If you are not sure, read the appropriate sections before making your selection.

☒ Life sciences ☐ Behavioural & social sciences ☐ Ecological, evolutionary & environmental sciences

For a reference copy of the document with all sections, see nature.com/authors/policies/ReportingSummary-flat.pdf

Life sciences study design

All studies must disclose on these points even when the disclosure is negative.

Sample size	According to our preliminary data, three mice in each group showed significant difference. Therefore, to assure the observation, we extend each group to 10 mice.
Data exclusions	No data were excluded from the analysis.
Replication	All attempts at replications were successful.
Randomization	Sample allocations were randomized.
Blinding	The administration of sevoflurane or propofol is impossible to be blind. The rest of data collecting including IVIS and nodule counting are objectively attained.

Reporting for specific materials, systems and methods

Materials & experimental systems

n/a	Involved in the study	
☒	☐	Unique biological materials
☐	☒	Antibodies
☐	☒	Eukaryotic cell lines
☒	☐	Palaeontology
☐	☒	Animals and other organisms
☒	☐	Human research participants

Methods

n/a	Involved in the study	
☒	☐	ChIP-seq
☒	☐	Flow cytometry
☒	☐	MRI-based neuroimaging

Antibodies

Antibodies used	CD11b (Abcam, ab133357), CD31 (55074), anti-p-Stat3 (Tyr 705, Cell signaling 9145; Ser727 Cell signaling 9134), anti-Stat3 (cell signaling 30835), anti-GAPDH (Santa Cruz sc-3233)
Validation	All antibodys are validated by manufacturers.

Eukaryotic cell lines

Policy information about cell lines

Cell line source(s)	4T1 and MDA-MB-231 stably expressing firefly luciferase were provided by Dr. Chia-Hsin Chan, Department of Pharmacological Science, Stony Brook University. 4T1 and MDA-MB-231 cell line were from ATCC.
Authentication	4T1 and MDA-MB-231 cell line were authenticated by short tandem repeat profiling by ATCC.
Mycoplasma contamination	4T1 and MDA-MB-231 cells were tested negative for mycoplasma contamination.
Commonly misidentified lines (See ICLAC register)	No commonly misidentified lines were used in this study.

Animals and other organisms

Policy information about studies involving animals; ARRIVE guidelines recommended for reporting animal research

Laboratory animals	6-week female NOD-SCID mice, 6-week female balb/c mice
Wild animals	The study did not involve wild animals.
Field-collected samples	The study did not involve animals collected in field.
